# Gelatine

**Published:** 1886-01

**Authors:** 


**Gelatine.**—Leibig, the renowned chemist, in speaking of gelatine as an article of food, says: "It has now been proved by the most convincing experiments that gelatine, by itself is tasteless, and when eaten excites nausea, possesses no nutritive value; that even when accompanied by the savory constituents of flesh it is not capable of supporting the vital powers, and when added to the usual diet as a substitute for plastic matter does not increase, but, on the contrary, diminishes the nutritive value of the food, which it renders insufficient in quantity and inferior in quality; and that its use is hurtful rather than beneficial. The only difference between it and joiners' glue is its greater price."

